# Insecticidal Management and Movement of the Brown Stink Bug, *Euschistus servus*, in Corn

**DOI:** 10.1673/031.011.16801

**Published:** 2011-12-09

**Authors:** Dominic D. Reisig

**Affiliations:** North Carolina State University, Department of Entomology, The Vernon James Research and Extension Center, 207 Research Station Road, Plymouth, NC 27962

**Keywords:** autocorrelation, Moran's /, transect

## Abstract

In eastern North Carolina, some brown stink bugs, *Euschistus servus* (Say) (Hemiptera: Pentatomidae) are suspected to pass the F_1_ generation in wheat (*Triticum aestivum* L.) (Poales: Poaceae) before moving into corn (*Zea mays* L.) (Poales: Poaceae). These pests can injure corn ears as they develop. To test their effectiveness as a management tactic, pyrethroids were aerially applied to field corn in two experiments, one with 0.77 ha plots and another with 85 ha plots. *Euschistus servus* population abundance was monitored over time in both experiments and yield was assessed in the larger of the two experiments. In the smaller experiment, the populations were spatially monitored in a 6.3 ha area of corn adjacent to a recently harvested wheat field (352 sampling points of 6.1 row-meters in all but the first sampling event). Overall *E. servus* abundance decreased throughout the monitoring period in the sampling area of the smaller experiment, but remained unchanged over time in the large-scale experiment. During all sampling periods in both experiments, abundance was the same between treatments. Yield was unaffected by treatment where it was measured in the larger experiment. In the smaller experiment, *E. servus* were initially aggregated at the field edge of the corn (two, six and 13 days following the wheat harvest). Sixteen days following the wheat harvest they were randomly distributed in the corn. Although it was not directly measured, stink bugs are suspected to move the cornfield edge as a result of the adjacent wheat harvest. More study of the biology of *E. servus* is needed, specifically in the area of host preference, phenology and movement to explain these phenomena and to produce better management strategies for these pests.

## Introduction

Stink bugs are well-documented itinerant pests of Southeastern field crops. Both the southern green stink bug, *Nezara viridula* L. (Hemiptera: Pentatomidae) and brown stink bug, *Euschistus servus* (Say) have been reported to injure field corn, *Zea mays* L. (Poales: Poaceae), in the late vegetative and early reproductive stages (Clower 1958; Negrón and Riley 1987; Blinka 2008; Ni et al. 2010). However, their potential to injure field corn beyond the V6 stage has not been documented or investigated as extensively as that for corn before the V6 stage (Clower 1958; Townsend and Sedlacek 1986; Sedlacek and Townsend 1988; Annan and Bergman 1988; Apriyanto et al. 1989a, 1989b). Past the V6 stage, stink bug injury potential is greatest before and at anthesis (VT-R2) (Negrón and Riley 1987; Ni et al. 2010). These data show that as corn ears develop and mature they become less sensitive to injury.

Within North Carolina, the brown stink bug, *E. servus*, and green stink bug, *Acrosternum hilare* Say, predominate (Barbour et al. 1990; Blinka 2008; Herbert et. al 2009). Both species are polyphagous, piercing, sucking pests that feed on many cultivated and noncultivated monocots and dicots (McPherson and McPherson 2000). In North Carolina, both species are bivoltine and overwinter as adults in organic residues and litter (Dietz et al. 1979; McPherson and McPherson 2000). Adults emerging in the spring sustain themselves and reproduce on various wild and cultivated hosts before the second generation moves to cultivated hosts (Dietz et al. 1979; Ehler 2000; McPherson and McPherson 2000; Blinka 2008). Stink bug movement and host preference in North Carolina largely remains a black box, with complex differences and host preferences observed, but seemingly inconsistent and unexplained. For example, in one year, *E. servus* adults were found in much higher abundance during three months of samples taken in field corn, compared to cotton *Gossypium hirsutum* L., peanut *Arachis hypogaea* L., soybeans *Glycine max* L., tobacco *Nicotiana tabacum* L., weeds (defined as ditch banks and pasture fields that contained both grasses and broadleaves), and wheat *Triticum aestivum*, in L. (Poales: Poaceae) late-planted soybean fields that were defined as wheat fields harvested in spring and double-cropped with soybean (Blinka 2008). The previous year, no trend was observed. In the statistical analysis, there was a significant interaction of *E. servus* abundance over sample year, time of sample within the growing season, and the crop samples that remained unexplored. Inconsistencies between crops and year of *A. hilare* abundance were also observed. Disentangling these complexities may prove challenging. Factors such as the interplay of plant phenology, weather, and plant host mosaic in the landscape, to name a few, may prove important in describing the biology and movement of these stink bugs.

*Euschistus servus* is able to colonize, feed and oviposit on a single host, wheat (Blinka 2008). The generation of *E. servus* that emerges from this host is then suspected to move into corn in eastern North Carolina. Although this movement has not been documented directly, *E. servus* abundance can increase in the edges of cornfields adjacent to wheat as the wheat grain matures (Blinka 2008). The piercing, sucking, peripatetic and polyphagous plant bug (*Lygus* spp.) is known to move into cotton in a similar fashion when a nearby, more preferable host is harvested (Stern et al. 1964; Sevacherian and Stern 1975; Fleischer et al. 1988). Wheat is typically harvested during early June in North Carolina, which often corresponds with the stage in field corn development in which the ear is forming, in the early stages of grain development. Corn is particularly susceptible to injury and damage during this period, when feeding may lead to reduced crop weight and abnormally shaped or deformed ears (Negrón and Riley 1987). Growers in North Carolina refer to this as “boomerang ear” or “banana ear.” These experiments were designed to test the effectiveness of aerial pyrethroid application for stink bug management and its impact on corn yield. Spatial movement of stink bugs in field corn was documented in one of the experiments where corn was adjacent to harvested wheat.

## Materials and Methods

### Spatial and small-scale insecticidal management study

An 80 ha cornfield, Z. *mays* was located near Columbia, NC that was bordered on three sides by wheat, *Triticum aestivum* L. (Poales: Poaceae). The corn hybrid was DKC6169 YieldGard VT Triple (DKC= Dekalb, Monsanto, Co., www.monsanto.com) and was planted on 7 April 2010 at 89,000 seeds/ha, with 91 cm wide rows. The previous 2009 crop was also corn. A wheat field adjoining the corn was harvested by the grower on 8 June 2010 and the stubble was burned on 10 June 2010.

**Figure 1.**
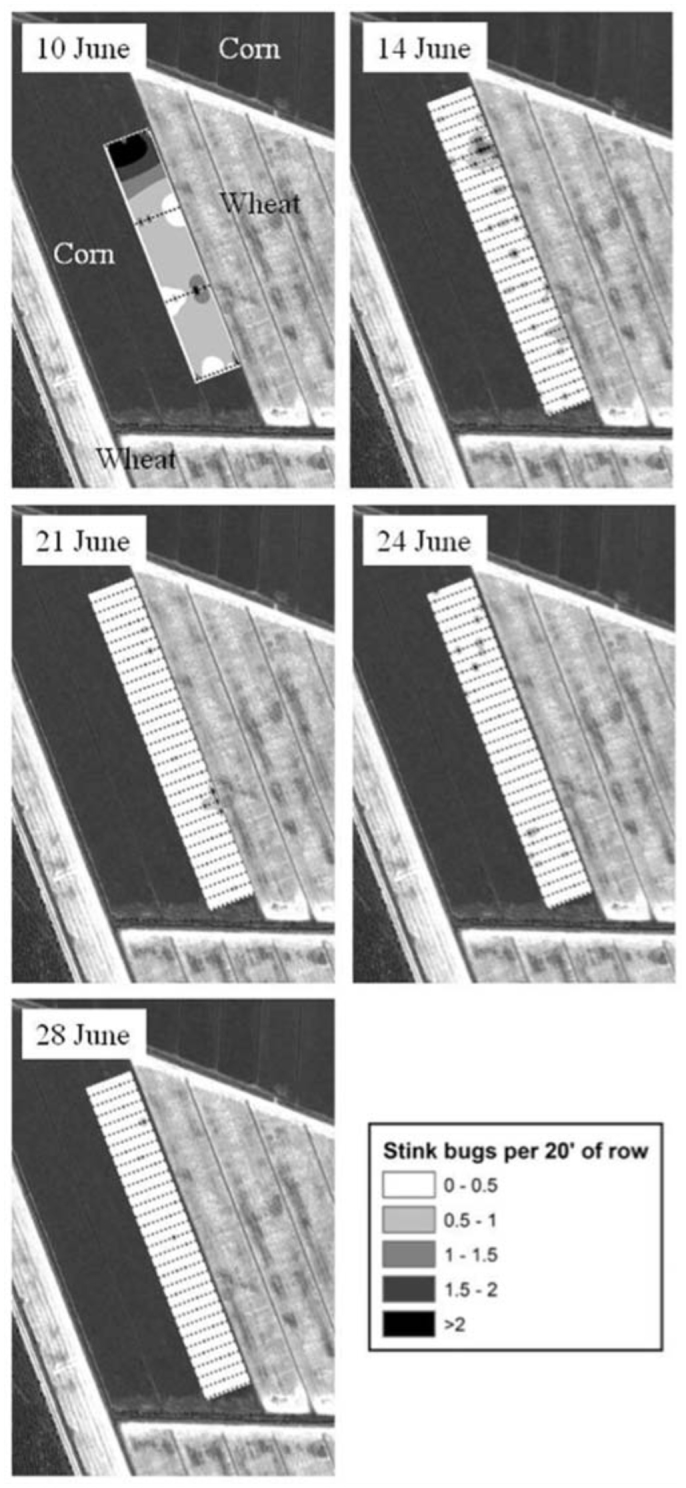
Spatial interpolation (inverse distance weighted using ArcView) of total stink bugs per 20 row-feet in Tyrrell County, NC corn sampling arena. Black circles represent sampling points. Surrounding crops notated in figure of first sampling. High quality figures are available online.

Corn was sampled for live stink bugs by searching individual plants along 6.1 m of row. Adult stink bug species and number was recorded. Egg masses and nymphs were recorded, but not identified to genus or species. On 10 June, there were four sample transects, extending from the edge of the corn adjacent to the field that previously contained wheat (Figure 1). These transects consisted of individual sampling points separated by 9.1 meters across the rows (the 11^th^ sampling location was 91 m from the border). Each of these sampling locations across the rows in the transect was paired with another corresponding sampling location in the next transect. These pairs were separated 171 m down an individual row (the fourth group of sampling locations was 512 m from the first). As a result, on 10 June, 44 total locations were sampled for stink bugs in the field, encompassing a 6.3 ha sampling area.

An aerial applicator was contracted to apply lambda-cyhalothrin (Karate Z at 33.6 g ai/ha, Syngenta Crop Protection, Inc., www.syngenta.com) in 21 m wide-passes on 11 June. Application volume was 28 L/ha; the corn stage was V12-V13 and it tasseled after V13. Alternating plots were created that were four spray-widths wide (85 m) by 91 m long. These treated plots were separated by untreated areas that were 85 m × 91 m. An aerial application (Karate Z at 33.6 g ai/ha in four 21m wide-passes) was also made on 21 June that was to overlap half of the treated area and half of the previously untreated area.

Stink bugs were again sampled from the 6.3 ha area as previously described on 14 June (3 days after the first treatment), 21 June (directly before the second treatment), 24 June (3 days after the second treatment) and 28 of June, as described above. However, instead of four transects of 11 sampling locations, the number was increased to 32 transects of 11 sampling locations (Figure 1). Each of these transects was separated by 21.3 m down the row, instead of the previous 171m.

Efficacy of the first insecticidal treatment was assessed through a repeated measures mixedmodels analysis of variance (ANOVA, PROCMIXED, SAS Institute 2008) approach. Fixed factors included treatment and the interaction of treatment and date. The REPEATED statement was used and the covariance was fit with a first-order autoregressive structure, based on the lowest X^*2*^ value criterion (Littell et al. 2006). A square root transformation was used to normalize the data and the GROUP= option was used in the REPEATED statement to correct the heteroscedasticity. Denominator degrees of freedom were calculated following the methods of Kenward-Roger (1997). Data included in this analysis were collected between 14 and 21 June. Data from the second insecticide treatment were not included in an ANOVA because the airplane did not spray the correct area. As a result, stink bug numbers are reported as an average for the field on 24 and 28 June. Untransformed means are presented as plus or minus standard error of the mean.

To test the degree of spatial dependence among the stink bugs sampled, data were projected in Arc View 9.3.1 (ESRI 2009). Global Moran's *I* was used to test for spatial autocorrelation among the region for each sampling date. Cocu et al. 2002 provide an excellent and detailed explanation of Gobal Moran's *I.* Briefly, Global Moran's *I* was calculated using the “Spatial Autocorrelation (Morans I)” spatial statistics tool in Arc View, with the equation defined as:


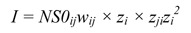


where the observations z*i*, z*j* are deviations from the mean and *wij* is the element in the spatial weights matrix corresponding to the observation pair *i*, *j. S*0 is the aggregate of all the spatial weights and *n* is the total number of features. The upper limit of the spatial weights matrix was defined as the area encompassing the entire sample arena. If *I* = 0, there was no spatial autocorrelation; if *I* > 0, the spatial autocorrelation was positive (surrounding sampling points had similar abundance); and if *I* < 0, the spatial autocorrelation was negative (surrounding sampling points had dissimilar abundance).

### Large-scale insecticidal management study

Corn was planted in four blocks near Beaufort, NC (Table 1) at 81,545 seeds/ha on 51 cm rows. On 25 June, lambda-cyhalothrin (Karate Z at 33.6 g ai/ha) was applied aerially, with 28 L/ha of water, to half of the north side of the four blocks, splitting them. The south portion remained untreated. Both the treated and untreated areas were approximately the same size and were approximately 85 ha in each replication (plot). Live adult stink bugs were sampled on 12 June, 22 June (both pretreatment) and 3 July (post-treatment). Presence of eggs and nymphs was noted, but their number was not recorded. When sampling preceded treatment, 20 plants in six random locations were searched for stink bugs in what was to become a treated area and groups of twenty plants in six random locations in each replication. The number of the twenty plant groups that were sampled at each location ranged from 2 to 4 on 12 June (average number was 2.6 ± 0.1, with a median of 3). On 22 June and 3 July, 4 to 6 groups of twenty plants were sampled at each location (average number was 4.8 ± 0.05, with a median of 5). The post-treatment sampling procedure was identical to the pre-treatment sampling. Yield was assessed using data from four John Deere 9750 STS combines (John Deere, Moline, IL), each equipped with an Ag Leader Technology PF3000 yield monitor (Ag Leader Technology, Ames, IA) and Ag Leader moisture sensing components. All four combines were used to harvest each plot and the area harvested by an individual combine ranged from 6.6 to 27.5 ha.

**Table 1.**
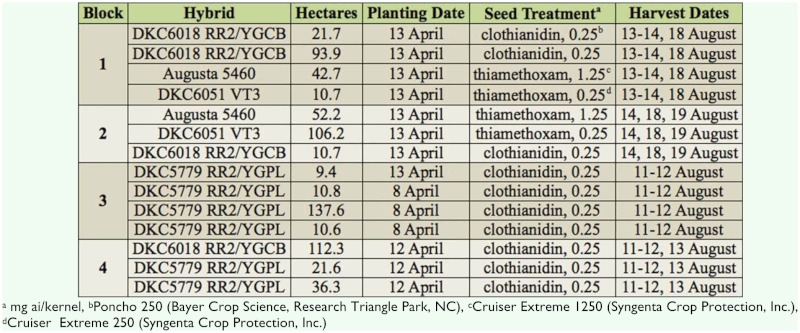
Corn hybrid 2010 planting and harvest information. DKC= Dekalb; RR2= glyphosate tolerant; YGCB= YieldGard Corn Borer (Monsanto, Co.); Augusta= Augusta Seed Corp., Mount Solon, VA; VT3= YieldGard VT Triple (Monsanto, Co.); YGPL= YieldGard Plus (Monsanto, Co.).

Mixed-models ANOVA (PROC MIXED) was used to compare yields between treatments. Yield data from each of the four combines was considered a pseudoreplicate and the mean yield for each plot was calculated using the yield from each of the four combines in each plot (two plots per block). Block was assigned as a random factor, while treatment was fixed. A repeated measures mixed-models ANOVA (PROC MIXED) approach was used to compare abundance over time. Fixed factors included treatment and the interaction of treatment and date. The REPEATED statement was used and the covariance was fit with a compound symmetry structure, based on the lowest *X^2^* value criterion (Littell et al. 2006). A square root transformation was used to normalize the data and the GROUP= option was used in the REPEATED statement to correct heteroscedasticity. Denominator degrees of freedom were calculated following the methods of Kenward-Roger (1997). Means were separated using Tukey's Honestly Significant Differences (Tukey's HSD) test to protect against Type I errors.

## Results

### Spatial and small-scale insecticidal management study

*Insecticidal management.* The average number of plants sampled at each 9.1 rowmeter sampling location during the study was 13.6 ± 0.1. *E. servus* was the dominant stink bug present, while *A. hilare* was not present. Other stink bugs (e.g. *Oebalus pugnax* F., *Proxys punctulatus* Pallisot and *E. tristigmus* (Say)) were not found in significant abundance (viz., *n* < 3 per sampling date). Stink bug abundance on 10 June, sampled one day prior to the aerial treatment, was 0.08 ± 0.23 stink bugs per plant. On 14 June, three days post-treatment, stink bugs per plant averaged 0.027 ± 0.007 in untreated plots and 0.019 ± 0.002 in treated plots. One week later, on 21 June, stink bugs per plant averaged 0.013 ± 0.005 in untreated plots and 0.006 ± 0.001 in treated plots. Stink bug abundance was not significantly different in treated and untreated plots (*F* = 2.26; d.f = 1, 6.95; *P* = 0.18), regardless of date, but they were significantly higher on 14 than 21 June (*F* = 11.69; d.f. = 1, 6.95; *P* = 0.01). After the second aerial treatment, stink bug abundance averaged 0.012 ± 0.001 per plant on 24 June (three days after the second application) and 0.008 ± 0.001 on 28 June.

**Spatial dynamics.** Stink bugs were significantly spatially autocorrelated (clustered) on 10 June (Moran's *I* = 0.10; Z = 2.3; *P* = 0.02), 14 June (Moran's *I* = 0.02; Z = 5.3; *P* < 0.0001) and 21 June (Moran's *I* = 0.02; Z = 4.3; *P* < 0.0001), but were randomly distributed on 24 June (Moran's *I* = 0.00; Z = 1.26 *P* = 0.21) and 28 June (Moran's *I* = 0.01; Z = -1.11; *P* = 0.27). When graphically depicted (Figure 1), clusters were visible in one end of the sampling area on 10 June and near the edge on 14 and 21 June.

### **Large-scale insecticidal management study**

**Figure 2.**
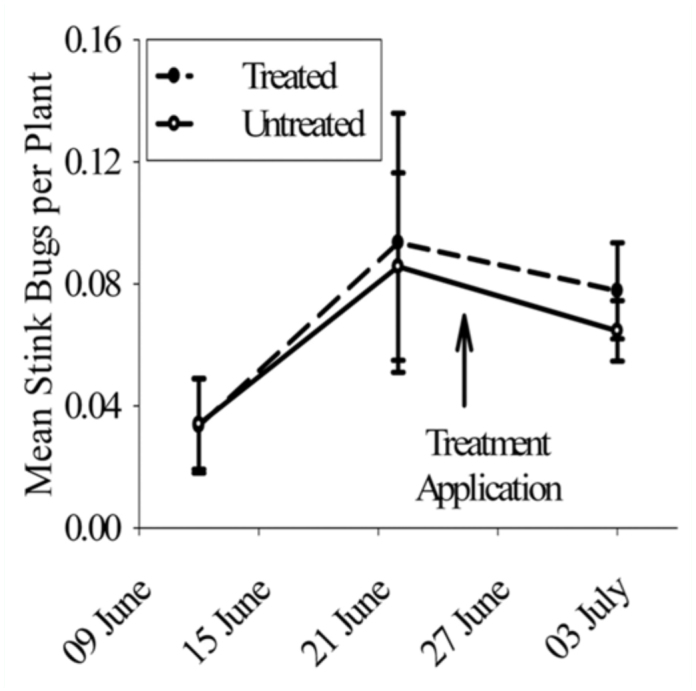
Mean *Euschistus servus* abundance per plant ± SEM preand post-treatment with lambda-cyhalothrin. Arrow represents application date. High quality figures are available online.

Eight days after treatment, stink bug abundance was the same between treated and untreated corn (Figure 2). Stink bug population abundance fluctuated significantly across sampling date (*F* = 5.47; df = 2, 6.1; *P* = 0.04), but the means were not statistically different using Tukey's HSD means separation procedure. Stink bug abundance was similar across dates among treatment (*F* = 1.12; df = 2, 8.19; *P* = 0.37) or treatment (*F* = 0.06; df = 2, 8.2; *P* = 0.82). Average yield was 391.1 ± 11.6 bu/ha in lambda-cyhalothrintreated plots and 404.1 ± 3.8 in untreated plots. They were not significantly different (*F* = 2.79; df = 1, 3; *P* = 0.19). Mean percent moisture was 20.1 ± 0.2.

## Discussion

Reduction in *E. servus* abundance from aerial lambda-cyhalothrin treatments was not observed in either experiment. Yield was the same between insecticide-treated and untreated field corn in the large-scale experiment. This is consistent with previous studies demonstrating that parameters associated with yield loss at this stage in corn development are less sensitive to injury (Clower 1958; Negrón and Riley 1987). Stink bugs were spatially clustered in the corn two, six, and 13 days after the wheat harvest in the adjacent field. Stink bugs were randomly distributed on later sampling dates. Most of these clusters occurred on the field edge of corn that was adjacent to the harvested wheat field. This provides further indirect evidence that *E. servus* may build up population abundance in wheat and move into adjacent corn.

*Euschistus servus*, the dominant species found in these experiments, is more tolerant to pyrethroids than organophosphates, as demonstrated by vial and field assays (Willrich et al. 2003). Consequently, tolerance could provide an explanation for lack of effective management. However, a small plot insecticide screening trial for stink bugs was initiated on 11 June 2010 in an area of the corn adjacent to the spatial and small-scale insecticidal management study (unpublished data). This trial was also adjacent to the harvested wheat and the location was chosen because stink bugs were relatively abundant. The insecticides for this study were applied one day after the aerial application for the small-scale spatial and insecticide management study in the same field. In the small-plot trial, an application of lambdacyhalothrin (Warrior II at 33.6 g ai/ha, Syngenta Crop Protection, Inc.) significantly reduced stink bug abundance by 93% in comparison to the check plots three days after treatment. Similar effects were seen using high rates of other pyrethroids (e.g., gammacyhalothrin and zeta-cypermethrin). Seven days after treatment there were no significant differences among treatments. Although tolerance to lambda-cyhalothrin cannot be ruled out as a contributing factor to the lack of effective stink bug management in these studies, it is unlikely given the significant effect observed in the small-plot experiment adjacent to the spatial and small-scale insecticidal management study.

Application methods between the small-plot experiment and aerially-applied insecticide experiments were markedly different. For example, the application volume of the small plot experiment was 75 L/ha compared to the 28 L/ha applied in the aerially-applied experiments. Moreover, a backpack sprayer fitted with a boom to spray above the corn was used and the application was made during the morning. As a result, stink bug abundance may have been effectively reduced using pyrethroids in the small-plot experiment due to differences resulting from the application method, such as increased coverage (i.e., that which might result from a higher application volume, different nozzles, reduced drift resulting from relative proximity to the crop canopy, etc.).

All insecticide applications were made during the morning; hence, the time of day probably did not have a major impact on the differences in the effect of insecticide on stink bug densities among experiments. Stink bugs were sampled in the morning and into the early afternoon. However, anecdotal differences in distribution among the plant were not observed throughout the day. Most stink bugs were observed hidden between the leaf collar and the stem of the plant. Aerial insecticidal management may be more effective if future research reveals that stink bugs have diurnal behavior in which they move from cryptic habitats to more exposed habitats.

Movement of stink bugs from untreated areas to treated areas is a possible explanation for the apparent lack of effective insecticidal management. The large-scale experiment was designed to eliminate this as a possible confounding effect, with plot sizes of 85 ha. Although the movement potential of stink bugs across the landscape is unknown, it seems unlikely that stink bugs would colonize and spread evenly throughout treated blocks eight days after treatment, when the posttreatment sample was taken. Stink bug abundance did not differ between treated and untreated areas at any point during the monitoring period in the spatial and smallscale experiment. However, stink bugs were clustered at the field edge, prior to, and three and seven days following the first pyrethroid treatment. As a result, it is unlikely that stink bugs moved from untreated areas into treated areas and more plausible that they were initially clustered at the edge of the cornfield because they had moved out of the adjacent harvested wheat field.

Stink bugs were spatially clustered in the corn two, six, and thirteen days after the adjacent wheat harvest. In Georgia, *N. viridula* and *E. servus* are known to be distributed toward the edge of corn, rather than the interior (Tillman 2010). Tillman 2010 reported data as a comparison between stink bug numbers in the edge of the field and those on the interior, lumped together over the entire field season. Their approach contrasts with the present study where stink bugs were sampled and spatially analyzed at different periods of time. In the present study, clusters were generally concentrated on the field edge, although possibly not on the sample taken two days after the wheat harvest. The sample taken two days after the wheat harvest was a “pretreatment” sample because it was taken one day before the aerial pyrethroid application. Sampling of the area was not as complete as the other dates and represented four transects of eleven longitudinal sampling points, instead of thirty-two transects of eleven longitudinal sampling points in the same area. Clusters on this day do not appear associated with the field edge, but this could be an artifact attributable to an insufficient number of sampling points. Burning of the wheat stubble, adjacent to the cornfield, was concurrent with the two day after harvest sample taken in the corn. The similar peripatetic, polyphagous, piercing, sucking pest *Lygus hesperus* moves in response to changes in light and wind (Blackmer et al. 2004; Bancroft 2005). Although movement was not directly observed, intransient stink bugs in the wheat stubble following harvest may have moved into the corn as a result of the burning.

Regardless, stink bugs were spatially clustered in the cornfield edge adjacent to the harvested wheat on six and thirteen days after the wheat harvest. These clusters were present after the first pyrethroid application and were most likely independent of it, since abundance was similar in treated and untreated areas. Stink bugs were randomly distributed after the second pyrethroid application. Although the aerial applicator did not spray the correct area in the second application, it is unlikely that this application affected stink bug abundance because they randomly distributed across the sampling area at three days after the second application (ten days after the first application). A more plausible explanation is that the stink bugs moved out of the sampling area toward the interior of the field, or out of the field. It is unclear whether stink bugs moved out of the study area as a result of this second application (e.g., due to the repellant properties of the pyrethroid (Elliot et al. 1978) or were independent of it.

The speed of movement of a peripatetic polyphagous insect through the vegetation is thought to provide an indirect measurement of host preference (Kareiva 1983; Bancroft 2005). The stink bugs in the study area were aggregated near the field edge up to thirteen days after the adjacent wheat was harvested. The number of stink bugs in the sample area decreased from the beginning to the end of the experiment. Stink bug abundance and range of calendar sampling date in the large-scale experiment was similar to that in the smallerscale experiment, but abundance did not change over time. It is unclear whether corn is a preferred host without a concurrent and direct comparison to the speed of movement through other crops. More work needs to be done to directly document stink bug movement and biology within and among crops to posit more substantial hypotheses.

In conclusion, aerial pyrethroid sprays for stink bug management in field corn are not effective to manage brown stink bug population abundance and will likely have little impact on yield, especially if they are applied after anthesis. If reduction of stink bug abundance using an insecticide is desired in the later vegetative stages of field corn when crop canopy height precludes the use of ground application, arial insecticide applications are necessitated by the height of the crop. Based on the movement data, if a ground-based application can be made to corn before crop canopy height precludes its use, it could be focused on the edge of corn after stink bugs have migrated from wheat and before they have dispersed into corn. Assuming this is a useful tactic, it is unclear when the exact time of this application should take place and how far it should extend into the field to be effective. Organophosphate insecticides are the most effective for *E. servus* (Willrich et al. 2003). With the loss of methyl parathion, there is not a highly effective insecticide for *E. servus* management registered for use on field corn in the Southeast. More research should focus on the biology of this pest to create an improved management strategy for use in field corn.
